# Three Dimensional Brain Reconstruction Optimizes Surgical Approaches and Medical Education in Minimally Invasive Neurosurgery for Refractory Epilepsy

**DOI:** 10.3389/fsurg.2021.630930

**Published:** 2021-09-27

**Authors:** Arun Swaminathan

**Affiliations:** Epilepsy Division, Department of Neurology, University of Nebraska Medical Center, Nebraska Medical Center Drive, Omaha, NE, United States

**Keywords:** brain reconstruction, minimally invasive surgery, refractory epilepsy, medical education, epilepsy surgery

## Abstract

Epilepsy is a prevalent condition that affects 1–3% of the population or about 50–65 million people worldwide (WHO estimates) and about 3.5 million people in the USA alone (CDC estimates). Refractory epilepsy refers to patients that respond inadequately to medical management alone (at least two anti-seizure medications at appropriate doses) and are appropriate candidates for other interventions such as brain surgery or the use of neurostimulators for their epilepsy. Minimally invasive techniques like stereotactic EEG electrodes offer excellent investigational abilities to study the diagnostic attributes of the seizure networks, while therapies like laser ablations and neurostimulators permit intervention and modulation of these networks to offer seizure control with minimal cognitive compromise and surgical morbidity. The accuracy of these techniques is highly contingent on precise anatomical correlation between the location of the electrodes and their proximity to relevant structures of the brain. Ensuring good anatomical correlation using 3-dimensional (3D) reconstructions would permit precise localization and accurate understanding of the seizure networks. Accurate localization of stereotactic electrodes would enable precise understanding of the electrical networks and identify vital nodes in the seizure network. These reconstructions would also permit better understanding of the proximity of these electrodes to each other and help confirm arrangement of neurostimulators to maximize modulatory effects on the networks. Such reconstructions would enable better understanding of neuroanatomy and connectivity to improve knowledge of brain structures and relations in neurological conditions. These methods would enable medical students and doctors-in-training to better their understanding of neurological disease and the necessary interventions.

## Introduction

Epilepsy affects about 1–3% of the population or about 50–65 million people worldwide (1), with an estimated patient population of 3.5 million in the USA (2). Seventy percent of patients achieve seizure control with the use of anti-seizure medications (3). Thirty percent of them remain poorly controlled despite the use of at least two medications at appropriate doses, qualify for a diagnosis of refractory epilepsy, and are appropriate candidates for further interventions such as epilepsy surgery, intracranial EEG studies and neuromodulatory devices (3). Epilepsy surgery represents one of the commonly performed interventions for such patients and can be performed using a variety of techniques. Procedures with higher invasiveness include intracranial EEG with grids and strips for diagnosis, and resections, lesionectomies and lobectomies for treatment. Minimally invasive procedures include stereotactic EEG electrode placement for diagnosis, and laser ablations or neurostimulators for treatment (some neurostimulators do offer diagnostic options as well). There has been increasing interest in the use of minimally invasive options over the more invasive ones, due to lower surgical morbidity from infections and hemorrhage and the ability to perform targeted interventions with ablations and neurostimulators, to minimize cognitive deficits and achieve good seizure control (4). Good surgical outcomes are contingent on accurate anatomic localization of seizure networks and EEG electrodes with respect to brain structures, to ensure accurate understanding and permit effective targeted intervention and modulation (5).

Reconstruction involves the use of presurgical and postsurgical scans. The presurgical MRI scans of the brain act as a template on which postsurgical CT scans with intracranial electrodes are superimposed to achieve accurate localization of electrode contacts vis-à-vis anatomical structures in the brain. Coregistration and reconstruction of brain structures is performed using software like Freesurfer or 3-dimensional (3D) Slicer to accurately reproduce neuroanatomy and enable surgical planning. Such digital reconstruction helps achieve precise spatial correlation between electrode locations and cerebral structures. Newer software upgrades also permit superimposition of brain mapping data from imaging studies or cortical stimulation procedures to achieve excellent anatomical, electrical, and clinical correlation in these patients. Such multimodal reconstructions and superimpositions generate a 3D topographic map that enables easy recognition of the seizure onset zone and overlap and margins between the seizure networks and eloquent cortical regions. Reconstruction is used by many centers for surgical planning and many different techniques are used across centers, albeit there is no single standardized protocol. Reconstruction can be used with all the commonly available surgical planning software, a feature that should exponentially increase its use across centers.

These digital and physical representations can be used in teaching anatomical and surgical techniques in addition to teaching cerebral anatomy and network characteristics of neurological disorders. In this article, we briefly elucidate some of these applications of 3D reconstruction to the field of epilepsy surgery and its use in surgical planning and medical education as well.

## Discussion

### Diagnostic and Therapeutic Options

Resective surgery, while effective in many cases, produces cognitive deficits, which necessitate the need to estimate risks and benefits accurately prior to proceeding with it. Minimally invasive surgical options permit good outcomes comparable to the more invasive ones, while permitting much better preservation of cognition in most cases. There is a growing tendency to use minimally invasive options for surgery whenever necessary, and only use the more aggressive options as a last resort.

Both types of procedures include diagnostic and therapeutic options. Minimally invasive diagnostic procedures include stereotactic EEG and the diagnostic aspects of neuromodulation, especially with the use of responsive neurostimulation systems. Therapeutic procedures include laser and ultrasound ablations, responsive neurostimulators, deep brain stimulators and disconnection procedures.

Highly invasive procedures include subdural grids and strips for EEG as diagnostic modalities, while resections or lobectomies and invasive surgeries like corpus callosotomies represent the therapeutic options.

Accurate anatomical correlation and structural analysis is essential for efficacious outcomes from all of these procedures, minimally invasive or otherwise. Coregistration of pre and post-surgical images using a variety of techniques to study the efficacy of surgery and plan surgeries accurately has been a consistent desire of physicians and surgeons for many years. Various techniques have been implemented and continue to be experimented upon to produce accurate imaging correlation permitting the highest degree of surgical accuracy and best estimates of surgical efficacy from such procedures.

### Role in Resective Procedures

Temporal lobectomies were among the earliest performed procedures and remain efficacious to this day with excellent surgical outcomes seen in appropriately selected patients. Modern techniques to improve on such surgeries have focused on accurate reconstruction and imaging correlation to permit good surgical outcomes. The use of such correlation techniques permits accurate localization of anatomical landmarks like Brodman's areas and resultant surgical planning to minimize volume of resected tissue (5). Bleeding complications often occur due to proximity of vascular structures to the surgical field. Three-dimensional reconstructions have permitted avoiding such vascular structures and resulted in minimizing blood loss and surgical morbidity (6).

Cognitive deficits from surgery often result due to loss of cortical gray matter and damage to subcortical white matter tracts as well. Diffusion Tractography Imaging (DTI) studies the locations of these white matter tracts and permits accurate estimations of their locations for surgical planning. Three-dimensional reconstructions of these images permit accurate surgical planning and minimize structural damage to white matter tracts and improves cognitive outcomes. DTI based reconstructions also permit identification and targeting of cerebral connections that are part of the seizure network but may not be obviously apparent on regular imaging. Targeting such structures would result in better surgical outcomes and minimize morbidity. The use of DTI to avoid Meyer's loop of the optic radiation to preserve visual fields in temporal lobe epilepsy surgery would be a classic example of such an application (7). Anecdotal evidence from various centers suggests that the use of reconstruction would enable precise resections with sparing of eloquent cortex and vascular structures, resulting in fewer complications, better targeting of the seizure onset zone and improved seizure reduction, resulting in overall improvements in surgical outcomes. Clinical studies supporting these observations have however, yet to be performed.

### Role in Diagnosis

Epilepsy surgery often relies on the use of an accurate estimation of a patient's seizure network to choose the most appropriate intervention for them. Localization of the seizure network often relies on multimodal presurgical investigations including MRI, PET, and other kinds of tests. The gold standard to localize the seizure network is however, intracranial EEG. Subdural grids and strips are the older method while stereotactic EEG is a relatively newer technique (8). Stereotactic EEG is minimally invasive and permits studying intracranial EEG from seizure networks over multiple lobes with much lower surgical morbidity than grids and strips. Lower rates of infection and hemorrhage, decreased rates of cerebral edema and the ability to study larger and subcortical regions of seizure networks, as compared to grids and strips, have greatly enhanced the attractiveness of stereotactic EEG for intracranial explorations (9). Bihemispheric explorations, subcortical structures like nodular heterotopias or other multilobar or diffuse pathologies like polymicrogyrias are excellent cases for exploration with stereotactic EEG, as many of these conditions are poorly or inaccurately visualized with the use of grids and strips resulting in incomplete or falsely localizing data. The ability to sample from deep sulcal structures and to combine stereotactic EEG with grids and strips for patients needing hybrid explorations makes this a highly versatile technique (10). Anatomical accuracy is paramount as the choice of therapeutic approaches is determined by the localization of seizure onset, spread and proximity to eloquent structures as well. Three-dimensional reconstructions of stereotactic EEG are necessary and serve to improve the efficacy of this technique (11).

Pre-surgical and post-surgical imaging and correlation are necessary to ensure prevention of electrode shift during EEG recording and functional mapping to preserve integrity of the data. Performing these reconstructions enables us to ensure that electrodes have not moved, or if they have moved, to account for these shifts and calibrate our imaging to plan our surgeries accurately (12). Excluding electrode shifts ensures that the seizure networks and functional maps are accurate and the appropriate intervention can be performed with minimal concern for removal of eloquent cortex and resultant cognitive deficits. It would also permit accurate positioning of neurostimulators for therapy for epilepsy, especially for those patients showing involvement of nonresectable eloquent cortex or targeting of minute structures like thalamic nuclei.

The use of robotic surgical assistants (ROSA) in implanting these electrodes accurately with minimal error has greatly contributed to the efficacy and accuracy of this technique and revolutionized diagnostic approaches in epilepsy surgical evaluations (13). The margins of placement error between desired and actual targets can be minimized to 0.85–3 mm using ROSA, which greatly enhances locational and diagnostic accuracy.

Precise coregistration and reconstruction would greatly enhance ability to localize seizure onset and spread, especially in conditions like bottom-of-sulcus dysplasias, which are highly amenable to focused intervention resulting in excellent surgical outcomes. Three-dimensional reconstructions would help define the seizure network better and enable placement of more intracranial EEG electrodes for better definition of the seizure network, if needed, resulting in narrowing down the seizure onset zone, thus offering amore limited field of surgery and a higher probability of precise targeting of the seizure network. Neuromodulation would also achieve better seizure control if the electrical stimulation fields were targeted better with accurate directional juxtaposition as well. While all these methods of improved diagnosis and therapy are expected to improve with the use of reconstruction, well-designed studies proving these findings are absent and need to be performed to offer clinically reproducible and valid evidence.

### Role in Ablations

Stereotactic laser ablation represents a relatively newer innovation in the field on minimally invasive epilepsy surgery that involves ablating tissue in the seizure onset zone or network using thermal effects of laser to cauterize said tissue. The best evidence supporting the efficacy of laser ablation to treat patients with epilepsy with curative intent comes from the ablation of structurally well-defined targets (14). It is also easily coupled with other minimally invasive epilepsy surgery techniques, most notably robotic stereo-encephalography. Highly invasive procedures like corpus callosotomies can now be performed using laser ablation, a development facilitated by the use of imaging reconstructions to determine surgical feasibility and enable surgical planning (15). Reconstructions are expected to offer a better understanding of three-dimensional representation of seizure networks, resulting in better decision making regarding the number of ablations needed in a given case and the directional approaches for said ablations, thus offering improved seizure reduction and minimizing surgical complications. Studies confirming these findings with scientific evidence, are however, lacking and need to be performed.

### Role in Neuromodulation

Responsive neurostimulator and deep brain stimulation systems represent a newer approach to the field of surgical epilepsy and involve implanting a generator battery attached to intracranial electrodes. These electrodes are able to identify and store seizures and enable electrical stimulation for treatment. Patient responses to the use of such responsive neurostimulators have been very good with excellent outcomes being reported in most cases (16). Accuracy of neurostimulators depends on radiological accuracy, especially when eloquent cortex or thalamic nuclei are involved. Three-dimensional reconstructions would permit accurate placement and directional positioning of electrodes, which would result in precise targeting of desired structures and configuration of electrical fields produced by the device, ensuring maximal efficacy. Reconstructional approaches would also offer the ability to use the same intracranial EEG electrode implantation pathway to place neurostimulators at the precise targets and avoid newer scar tissue formation from creating a new pathway to implant the stimulator. While there are no clinical trials studying the comparative results from different spatial or electrical configurations of device, it stands to reason that finding the most optimal configuration and direction of the electrodes and resultant magnetic fields would maximize the therapeutic benefit from the device.

### Role in Teaching

Teaching complex concepts of neurophysiology to trainees and practicing physicians requires a deeper understanding of structural connectivity in three-dimensional space. The use of interactive reconstruction would greatly improve understanding of such concepts relevant to surgical epilepsy. It would promote individualized approaches to surgeries, especially in patients with variant anatomy or prior surgical intervention. The use of reconstructions permits us to guide and instruct trainees or physicians to improve their planning and surgical skills. Similar approaches are already underway concerning teaching and performing surgeries like disconnections or resections (17). Improved efficacy and user friendliness of such software and techniques will result in their greater use and consequent improvements in anatomical and surgical planning. Dedicated educational research studies are necessary to quantify and understand the long-term impact of reconstruction in teaching and research design.

### Role in Changing Approaches to Epilepsy

Reconstruction has produced change in our understanding and approach to the evaluation of epilepsy patients and syndromes as a whole. Diagnostic approaches are determined by the nature of imaging and quality of available EEG data to understand the seizure network and design an appropriate treatment paradigm for individual patients as well.

Reconstruction has necessitated the need for higher definition in medical imaging resulting in improved detection rates of brain anomalies and increased abilities to target these regions of the brain and confirm their involvement in seizure networks. There has been an increase in publications showing the efficacy of higher definition imaging and reconstruction in identifying brain abnormalities, higher rates of intervention and modification in surgical planning, better targeting with stereotactic EEG and resultant improvement in surgical outcomes and seizure reduction (18, 19). Previously non-lesional patients are now recognized as having subtle lesions, many of which are epileptogenic; while the pathogenicity of some lesions and malformations is still being understood. Reconstruction has contributed to an improved rate of lesion detection and increased understanding of seizure networks. Subtle lesions like cortical dysplasias, polymicrogyrias, heterotopias are more likely to be detected, explored and successfully intervened upon to achieve better cognitive outcomes and seizure reduction.

Stereotactic EEG studies are able to localize and identify nodes in a seizure network with increasing accuracy enabling precise targeting for resection or laser ablation. Focal epilepsies arising from or adjacent to eloquent cortex can be intervened upon with great precision using reconstruction. This is especially useful when these reconstructions are combined with electrographic data from brain mapping to delineate margins of resection or identify regions of overlap between epileptogenic cortex and eloquent cortex. Physicians are able to use this data to determine amount of tissue to be resected and targets for neurostimulation using responsive neurostimulators for treatment. We have expanded the number of patients that can be intervened upon and improve seizure reduction, tremendously changing the landscape of epilepsy surgery.

Bitemporal epilepsy has had significant changes in management resulting from reconstruction and neurostimulation. Current practices include presurgical testing followed by responsive neurostimulation over both temporal lobes to capture chronic electrocorticography from both sides to make an informed decision about the need for a unilateral temporal lobectomy or laser ablation (20). Reconstruction has significantly improved our ability to implant stereotactic electrodes and neurostimulators, in addition to increasing our understanding of mesial and neocortical temporal epilepsies, along with temporal plus epilepsy syndromes.

Multifocal epilepsies remain a challenging group of conditions to define and treat, but reconstructive approaches have improved our diagnostic efficiency and expand our therapeutic surgical options. Combined use of ablations or resections with neurostimulation to target the most active nodes in the seizure network is expected to improve seizure reduction rates in such syndromes, although scientific studies on these are yet to be published. Some studies showing targeted intervention at nodules in tuberous sclerosis with resultant improvement have been published (21–23), and it follows that reconstruction would produce improved definition of lesions and more efficacious surgical planning and intervention.

Generalized epilepsies remain a challenging group of syndromes to treat due to their diverse network properties and refractoriness to anti-seizure medications. The use of deep brain stimulation for movement disorders like essential tremor or Parkinson's disease is well-known and these concepts were extrapolated to the management of epilepsy as well. The US FDA approved deep brain stimulation of the anterior nucleus of thalamus for the treatment of refractory epilepsy following results from the SANTE trial (24). Responsive neurostimulation targeting thalamic structures (anterior, centromedian and dorsomedial nuclei) for refractory focal or multifocal or generalized epilepsies has also been performed with promising outcomes (25, 26). Reconstruction and well-defined imaging play a prominent role in targeting of these minute thalamic structures and resultant treatment of refractory epilepsies, generalized, or focal/multifocal. More studies are needed to determine the value of reconstruction in determining direction of stimulators and their electrical fields in these syndromes.

### Basic Principles of Reconstruction

**Figure 1:**
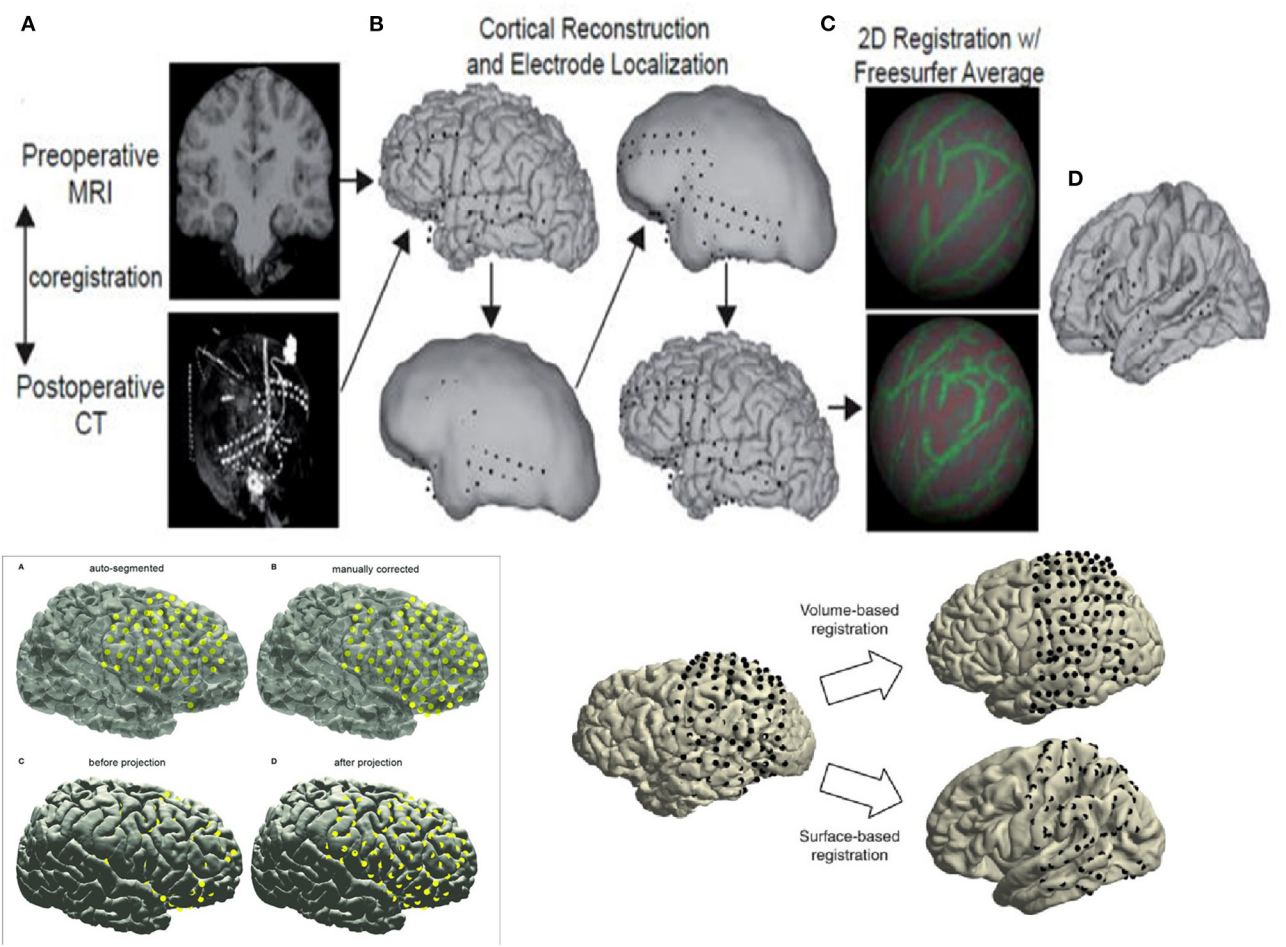
Basic principles of reconstruction. **(A)** Left hand Top corner - Preoperative MRI and postoperative CT scan are superimposed to produce the reconstructions. **(B)** Top Center - Electrode contacts and Brain Cortex are recreated to design the reconstruction accurately. **(C)** Right hand Top corner - Reconstruction is begun using software like Freesurfer to reconstruct brain in 2 dimensions before collation into 3 dimensions. **(D)** Right extreme Top corner - Reconstructed images with superimposed electrode contacts are seen at the end. Left hand Bottom corner - Images **(A–D)** show the principles of reconstruction using software and manual corrections - automatic segmentation of imaging followed by manual correction proceeding to preliminary reconstructions followed by final reconstruction with smoothing out of images and confirmation of cortical anatomy. Right hand Bottom corner - Images showing the use of the 2 main techniques in reconstruction - volume based recon vs cortical surface anatomy based recon.

**Figure 2:**
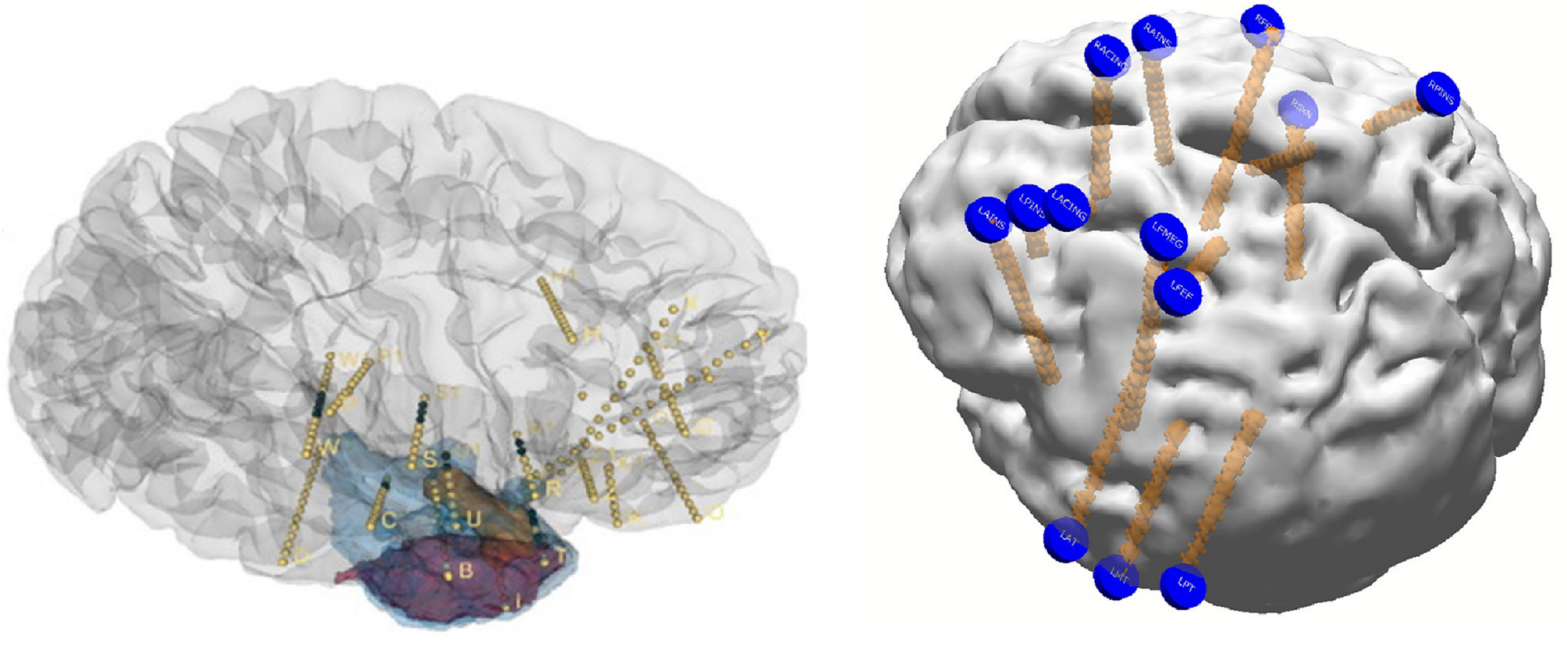
Samples of reconstructed images from two different patients.

Reconstruction involves using preoperative MRI scans of the brain and postoperative CT scans to precisely identify structures and recognize their relative position with respect to EEG electrodes with supporting roles played by reconstruction software. While the precise details are beyond the scope of this review, basic principles are outlined in Figure 1. Reconstruction produces an accurate topographical map of the brain and the placed electrodes and a sample image from our institution has been shown in Figure 2. It can be used during the exploratory phase to study localization of stereotactic or subdural electrodes and during the treatment phase to plan resections or neurostimulator implants as well.

## Conclusions

Diagnostic and therapeutic approaches are constantly being redefined to meet ever-increasing standards of efficacy and cognitive preservation to permit the best possible quality of life for patients. Three-dimensional reconstructions permit accurate anatomical and electrographic correlation enabling precise localization of seizure networks and functional mapping resulting in tailored and accurate intervention to maximize surgical outcomes and seizure control in patients with the least amount of cognitive compromise. The use of such techniques for teaching trainees is paramount and beneficial to the field of epilepsy surgery as a whole. Improved quality and innovations in such techniques are expected to advance the field and are a continuous source of interest to clinicians and academics alike.

## Author Contributions

The author confirms being the sole contributor of this work and has approved it for publication.

## Conflict of Interest

The author declares that the research was conducted in the absence of any commercial or financial relationships that could be construed as a potential conflict of interest.

## Publisher's Note

All claims expressed in this article are solely those of the authors and do not necessarily represent those of their affiliated organizations, or those of the publisher, the editors and the reviewers. Any product that may be evaluated in this article, or claim that may be made by its manufacturer, is not guaranteed or endorsed by the publisher.
